# Immunisation coverage and its determinants among children aged 12-23 months in Atakumosa-west district, Osun State Nigeria: a cross-sectional study

**DOI:** 10.1186/s12889-016-3531-x

**Published:** 2016-08-30

**Authors:** Elizabeth B. Adedire, Ikeoluwapo Ajayi, Olufunmilayo I. Fawole, Olufemi Ajumobi, Simon Kasasa, Peter Wasswa, Patrick Nguku

**Affiliations:** 1 Nigeria Field Epidemiology and Laboratory Training Programme, Abuja, Nigeria; 2 LAUTECH Teaching Hospital, Osogbo, Osun State Nigeria; 3 Epidemiology and Medical Statistics Department, University of Ibadan, Ibadan, Nigeria; 4 School of Public Health, Makerere University College of Health Sciences, Kampala, Uganda; 5 African Field Epidemiology Network, Plot 42, Lugogo By-Pass, Kampala, Uganda; 6 National Malaria Elimination Programme, Federal Ministry of Health, Abuja, Nigeria

**Keywords:** Immunisation coverage, Children, Rural district, Southwest Nigeria

## Abstract

**Background:**

Routine immunisation (RI) contributes immensely to reduction in mortality from vaccine preventable diseases (VPD) among children. The Nigerian Demographic and Health Survey, 2008 revealed that only 58 % of children in Osun State had received all recommended vaccines, which is far below World Health Organization (WHO) target of 80 %. We therefore, assessed RI uptake and its determinants among children in Atakumosa-west district of Osun State.

**Methods:**

Atakumosa-west district has an estimated population of 90,525 inhabitants. We enrolled 750 mothers of children aged 12–23 months in this cross-sectional study. Semi-structured questionnaires were used to obtain data on socio-demographic characteristics, knowledge of mothers on RI, history of RI in children and factors associated with full RI uptake. A fully-immunised child was defined as a child who had received one dose of Bacillus-Calmette-Guerin, three doses of Oral-Polio-Vaccine, three doses of Diptheria-Pertusis-Tetanus vaccine and one dose of measles vaccine by 12 months of age. We tested for the association between immunisation uptake and its likely determinants using multivariable logistic regression at 0.05 level of significance and 95 % confidence Interval (CI).

**Results:**

Mean ± (SD) age of the mothers and children were 27.9 ± 6.1 years and 17.2 ± 4.0 months, respectively. About 94 % (703/750) of mothers had received antenatal care (ANC) and 63.3 % (475) of the children possessed vaccination cards. Seventy-six percent (571/750) had good knowledge of RI and VPD. About 58 % (275/475) of children who possessed vaccination card were fully-immunised. Mothers antenatal care attendance (aOR = 3.3, 95 % CI = 1.1-8.3), maternal tetanus toxoid immunisation (aOR = 3.2, 95 % CI = 1.1-10.0) access to immunisation information (aOR = 1.8, 95 % CI = 1.1-2.5) and mothers having good knowledge of immunisation (aOR = 2.4, 95 % CI = 1.6-3.8) were significant determinants of full immunisation.

**Conclusions:**

Routine immunisation uptake was still below WHO target in the study area. Encouraging mothers to attend antenatal care and educational interventions targeted at rural mothers are recommended to improve vaccination status of children in the rural communities.

**Electronic supplementary material:**

The online version of this article (doi:10.1186/s12889-016-3531-x) contains supplementary material, which is available to authorized users.

## Background

Immunisation remains one of the most cost-effective public health strategies to reduce morbidity and mortality associated with vaccine preventable diseases. Routine immunisation (RI) has contributed immensely to significant reduction in mortality from these vaccine preventable diseases among children. Globally, it is estimated that about two to three million deaths occurs yearly as a result of vaccine preventable diseases (VPD) with approximately 1.5 million deaths among under-five children [1].

The World Health Organisation (WHO) established the expanded programme on immunisation (EPI) in 1974, with the goal of ensuring full accessibility of routine immunisation vaccines to all children. According to the EPI, a child should receive Bacillus Calmette Guerin (BCG), three doses of oral polio vaccine (OPV) and Diptheria Pertusis Tetanus (DPT), and measles vaccines by 12 months of age to ensure maximum protection against VPDs. Receipt of these vaccines at the recommended ages and intervals will provide the children adequate protection from VPDs [2].

Despite significant gains with routine immunisation coverage over the years, millions of children living in developing countries are not fully immunised, exposing them to disabilities or premature death. In 2014, over 60 % of the 18.7 million infants who were not fully vaccinated lived in ten countries including India, Nigeria, Democratic Republic of Congo, Ethiopia, Iraq, Pakistan, Philippines, Indonesia, Uganda and South Africa [1].

In Nigeria, VPDs are attributable for 22 % of childhood deaths amounting to over 200,000 deaths per year [3]. Routine immunisation coverage has remained low in many parts of Nigeria. Even though vaccines are provided free of charge by the government, coverage rates for routine immunisation antigens in many parts of Nigeria still fall below 50 % [4–7]. According to the 2008 Nigerian Demographic and Health Survey Report, only 57.8 % of children aged 12–23 months in Osun State were fully immunised, far below the WHO target of 80 % [8]. The situation is worse in the rural areas as children in these areas were twice less likely to receive full doses of RI vaccination than those in urban areas [8]. Studies have shown that uptake of immunisation services depends not only on provision of these services but other factors related to maternal knowledge, geographical accessibility and many other socio-demographic characteristics [8–10].

Rural communities represent highly marginalised areas in terms of distribution and access to health care interventions including immunisation services. This underscores the need to determine immunisation coverage rates and the factors influencing uptake of RI services in these marginalised areas. Thus, we conducted a study to assess immunisation coverage rates and to identify the factors associated with vaccination status of children 12–23 months in a rural district in south-western Nigeria.

## Methods

### Study setting

We conducted this study in Atakumosa-west district which is a predominantly rural district in Osun State, south-western Nigeria. It had an estimated population of 90,525 inhabitants based on the 2007 population census [11]. Atakumosa-west district is made up of eleven wards with about 170 widely distributed settlements. The Yorubas are the main ethnic tribe residing in the area most of whom are farmers. There are 27 primary health centers and two comprehensive health centers all of which provide routine immunisation.

### Study design

We conducted a community-based cross-sectional study between September and October 2013. Mothers of children 12–23 months old who were resident in the district at the time of the survey were interviewed. At the time of the study, children 12–23 months of age were considered eligible for sampling.

### Sample size determination

We used the method in the WHO immunization coverage cluster survey reference manual to determine the sample size based on a full immunisation coverage of 57.8 % [9], significance level of 5 % corresponding to a standard normal deviate (z) of 1.96, precision of 5 % and design effect (DEFF) of 2 and obtained a minimum sample size of 750 children [12].

### Sampling procedure

We used a two-stage cluster sampling technique to sample eligible children. At stage one (selection of clusters), we selected 30 clusters from the available 170 clusters based on probability- proportional- to- size of the population. In stage two (selection of households), we selected 25 households from each of the 30 clusters selected at stage one. The first household in each cluster was selected randomly and subsequent households were selected contiguously in the right direction until the required number of households for that cluster was achieved. From each selected household, one eligible child was selected. If a selected household had more than one eligible child, only one was randomly selected. If a selected household had no eligible child, the next contiguous household was visited and one eligible child selected. We sampled an equal number of children from each of the 30 clusters [12]. Thus, 25 children were sampled per cluster, giving a total sample size of 750 children.

### Data collection

Data for the study were collected by 15 trained community health extension workers using standardised structured and pretested interviewer-administered questionnaires. The questionnaires were administered in ‘Yoruba’; the predominant spoken language and back translated to English to avoid any ambiguity. Data collected include socio-demographic characteristics of mothers and children, knowledge of mothers regarding routine immunisation, vaccination status of children and reasons for incomplete or non-vaccination. If a card was available, the interviewer recorded the vaccination information and dates of each vaccination received by the child. If a child had never received a vaccination card, or the mother was unable to show the card to the interviewer, the vaccination information for the child was based on the mother’s report.

### Knowledge of respondents on RI and VPDs

To assess the knowledge of mothers, responses were scored using six questions on various aspects of routine immunisation. The questions assessed respondent’s ability to state: the correct purpose of immunisation, correct age a child should receive second dose of RI vaccinations, last dose of RI vaccines, total number of visits a child should make to the health facility to receive all recommended doses, at least three symptoms of vaccine preventable diseases and at least three vaccine preventable diseases. Each correct response was scored one point while each wrong response was scored zero. Mothers who scored three points and below were graded as having poor knowledge while those who scored four points and above were graded as having good knowledge. This scoring system is similar to that used in determining vaccination coverage in Nigeria [9].

### Outcome variable

#### Vaccination status of children

Based on the type and doses of RI antigens received, we categorized the children as fully immunised, partially immunised, or un-immunised. We defined a “fully immunised child” as a child who had received one dose of BCG, three doses of OPV (excluding OPV given at birth), three doses of DPT vaccine and one dose of measles vaccine by 12 months of age; “partially immunised child” a child who missed at least any one of the above doses; “un-immunised child” a child who had not received any vaccine by 12 months of age [13].

### Data analysis

Data were entered, cleaned and edited for inconsistencies before analyzing with Epi info version 7. Descriptive analysis was done and the results were summarized as frequencies and proportions for categorical variables and means and standard deviations (SD) for continuous variables. During bivariate analysis, associations between categorical variables were assessed using the Chi square test at 95 % Confidence Interval (CI). A multivariable logistic regression model with full immunisation status as dependent variable was built to rule out possible confounders. All analyses were done at 95 % CI and 0.05 level of significance.

## Results

### Socio-demographic characteristics of mothers and children

A total of 750 mothers were interviewed with mean age (SD) of 27.9 (6.1) years. Fifty-five percent of mothers were 20 – 29 years old, 3.1 % had no formal education, and 94.1 % were married. The mean age (SD) of the children was 17.2 (4.0) months; age of children ranged from 12 to 23 months and about half (50.4 %) of the children were males (Table 1).

**Table 1:** Socio-demographic characteristics of mothers and children in rural Atakumosa-west district, Osun State, Nigeria- September 2013

Variables/characteristics	Frequency (*n* = 750)	Proportion (%)
Age-group (years)		
< 20	39	5.2
20–29	415	55.3
30–39	251	33.5
≥ 40	45	6.0
Education		
No formal education	23	3.1
Completed primary	172	22.9
Completed secondary	402	55.3
Post secondary	153	20.4
Marital Status		
Married	706	94.1
Single	27	3.7
Divorced/widowed	17	2.2
Children age-group (months)		
12–15	133	23.5
16–19	170	30.1
20–23	262	46.4
Sex of child		
Male	372	49.6
Female	378	50.4
Childs’ birth order		
1	233	31.3
2	216	29.0
≥ 3	296	39.7
Place of birth		
Health facility	611	81.5
Non-health facility	139	18.5

### Vaccination coverage rate

The fully vaccinated coverage rate obtained by mothers’ recall was 74.4 % and fully vaccinated coverage rate obtained by immunisation card for all the antigens was 57.9 %. The proportion of children vaccinated with antigens given at birth and six weeks of age were more than the proportions of children vaccinated with antigens given at later ages (Table 2).

**Table 2:** Vaccination coverage for routine immunisation antigens in rural Atakumosa-west district, Osun State, Nigeria-September 2013

Routine immunisation antigens	Coverage by mothers’ recall *n*, (%); [95 % CI] *n* = 750	Coverage by immunisation card *n*, (%); [95 % CI] *n* = 475
Antigens administered at birth		
BCG	687 (91.6) [89.3 – 93.4]	406 (85.5) [81.9 – 88.5]
OPV 0	693 (92.4) [90.2 – 94.2]	395 (83.2) [79.4 – 86.4]
HBV 1	677 (90.3) [87.9 – 92.3]	392 (82.5) [78.7 – 85.7]
Antigens administered at 6 weeks		
OPV 1	681 (90.8) [88.5 – 92.3]	386 (81.3) [77.4 – 84.6]
DPT 1	694 (92.5) [90.4 – 94.3]	400 (84.2) [80.5 – 87.3]
HBV 2	676 (90.1) [87.7 -92.1]	382 (80.4) [76.5 – 83.8]
Antigens administered at 10 weeks		
OPV 2	655 (87.3) [84.7 – 89.6]	362 (76.2) [72.1 - 79.9]
DPT 2	659 (87.8) [85.3 – 90.1]	370 (77.9) [73.8 – 81.5]
Antigens administered at 14 weeks		
OPV 3	636 (84.8) [81.9 – 87.3]	339 (71.4) [67.0 – 75.4]
DPT 3	638 (85.1) [82.3 – 87.5]	352 (74.1) [69.9 – 77.9]
HBV 3	615 (82.0) [79.0 – 84.7]	348 (73.3) [69.0 – 77.1]
Antigens administered at 9 months		
Measles	622 (82.9) [80.0 – 85.5]	317 (66.7) [62.3 – 70.9]
Yellow fever	597 (79.6) [76.5 – 82.4]	311 (65.5) [60.9 – 69.7]
% Fully vaccinated	558 (74.4)	275 (57.9) [53.3 – 62.4]

### Vaccination status of children 12–23months

**Fig. 1:**
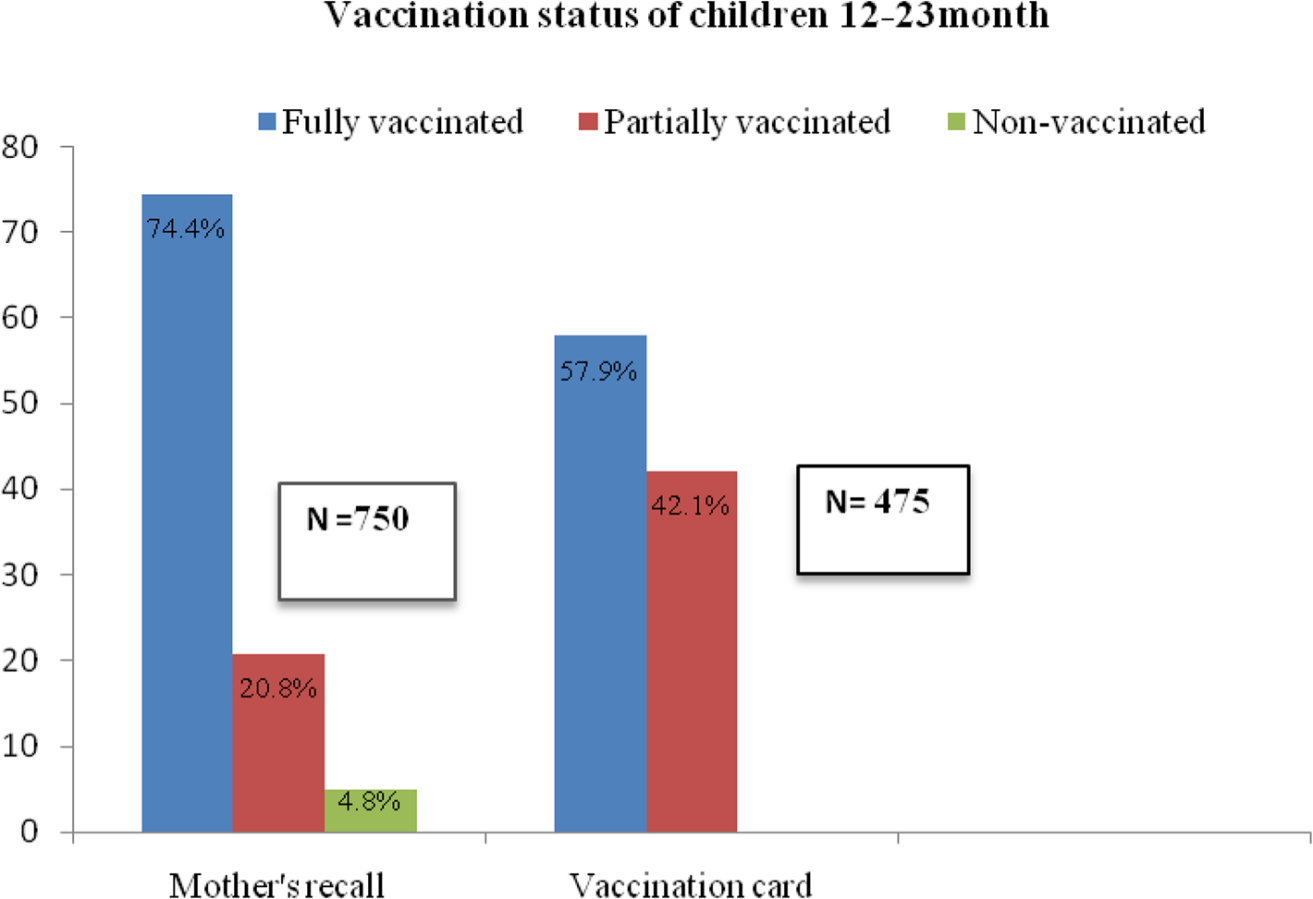
Vaccination status of children 12-23 months in rural Atakumosa-west district, Nigeria- September 2013

Of the 750 children, 475 possessed vaccination cards, indicating a vaccination card retention rate of 63.3 %. As shown in Fig. 1. Using mothers’ recall, 558 (74.4 %) of the children were fully-vaccinated, 192 (20.8 %) were partially-vaccinated, while 36 (4.8 %) were non-vaccinated. However, based on immunisation cards, 275 (57.9 %) of the children were fully immunised while 200 (42.1 %) were partially immunised (Fig. 1). On comparison of the mothers assessment of completeness of vaccination with card assessment using Kappa’s statistics, the number of observed agreements was 331.0 (69.7 % of the observations) and the number of agreements expected by chance was 258.3 (54.4 % of the observations), giving a kappa of 0.336 [SE of kappa = 0.041, 95 % CI; 0.256 - 0.416]. The agreement between the mothers assessment and card assessment was low, thus further analysis were done using information from the cards.

### Determinants of immunisation status

Table 3 shows the bivariate and multivariate analysis of factors associated with full immunisation status using the information obtained from the vaccination cards. Five factors namely; mothers age >35 years, antenatal care (ANC) visits by mother, maternal tetanus toxoid immunisation, maternal knowledge of routine immunisation and access to immunisation information in last 12 months were significantly associated with full immunisation in bivariate analysis; however following multivariate logistic regression; mothers who accessed ANC (aOR = 3.3, 95 % CI = 1.2-8.3), mothers who had at least one dose of tetanus toxoid immunisation (aOR = 3.2, 95 % CI = 1.1-10.0), mothers having good knowledge of immunisation (aOR = 2.4, 95 % CI = 1.6-3.8) and having access to immunisation information in the last 12 months (aOR = 1.8, 95 % CI = 1.1-2.5) were significant predictors of full immunisation status.

**Table 3:** Determinants of immunisation status of children 12-23 months in rural Atakumosa-west district, Osun State, Nigeria-September 2013

Variables	Fully immunised *n* = 275	Not fully immunised d *n* = 200	cOR [95 % CI]	*p*-value	aOR [95 % CI]	*p*-value
Age of mother (years)						
< 35	238 (60.2)	157 (39.8)	1		1	
≥ 35	37 (46.3)	43 (53.7)	1.8 [1.1 – 2.9]	**0.02**	1.1 [0.9 – 1.3]	0.15
Education						
No	64 (53.3)	56 (46.7)	1			
Formal/primary	211 (59.4)	144 (40.6)	0.8 [0.5 – 1.2]	0.24		
Secondary/higher						
Child sex						
Female	134 (55.8)	106 (44.2)	1			
Male	141 (60.0)	94 (40.0)	0.7 [0.6 – 1.2]	0.35		
Child birth order						
≥ 3	106 (55.2)	86 (44.8)	1			
< 3	169 (59.7)	114 (40.3)	0.8 [0.5 – 1.1]	0.32		
Place of birth						
Health facility	235 (59.1)	163 (40.9)	1			
Home/TBA^†^	40 (51.9)	37 (48.1)	1.3 [0.8 – 2.2]	0.25		
Antenatal care						
No	7 (33.3)	14 (66.7)	1		**1**	
Yes	268 (59.1)	186 (40.9)	2.8 [1.1 – 8.1]	**0.02**	**3.3 [1.2 – 8.3]**	**0.03**
Health facility within 5 km to residence						
Yes	248 (59.1)	172 (40.9)	1		1	
No	27 (49.1)	28 (50.9)	0.7 [0.4 – 1.2]	0.16	1.6 [0.9 – 2.8]	0.11
Maternal tetanus toxoid						
None	5 (27.8)	136 (72.2)	1		**1**	
At least a dose	270 (59.1)	187 (40.9)	3.8 [1.3 – 1.7]	**0.001**	**3.2 [1.1 – 10.0]**	**0.04**
Maternal knowledge on RI						
Poor	40 (40.4)	59 (59.6)	1		**1**	
Good	235 (62.5)	141 (37.5)	2.4 [1.5 – 3.8]	**<0.001**	**2.4 [1.6 – 3.8]**	**0.00**
Access to immunisation information in last 12 months						
No	71 (68.3)	33(31.7)	1		**1**	
Yes	204 (54.9)	167(45.1)	1.8 [1.1 – 2.9]	**0.01**	**2.5 [1.1 – 2.5]**	**0.02**

TBA^†^: Traditional birth attendant

Text in bold denotes statistically significant results

## Discussion

We assessed the immunisation coverage rate for routine immunisation and its determinants in rural district of Atakumosa-west Nigeria. The coverage rates for antigens given at birth (BCG, OPV0, and Hepatitis B Virus 1 [HBV]) were relatively higher compared with those given at later ages (measles and yellow fever vaccines). Based on mothers recall, 74.4 % of the children were fully vaccinated while only 57.9 % of children were fully vaccinated based on immunisation card assessment. We found that more than three-quarters of the mothers of children 12 – 23 months had good knowledge on routine immunization; however misconception still existed among a few. The factors found to positively influence completion of recommended schedule of routine immunisation included mothers’ attendance of antenatal care, mothers receipt of at least one dose of tetanus toxoid immunisation, mothers having good knowledge of immunisation and having access to information on routine immunisation.

Mothers are influential and known to play important roles in the vaccinations of their children. Majority of the mothers had good knowledge, which is similar to findings in rural south-western Nigeria which showed that about four-fifth of respondents had good knowledge on routine immunisations [9]. About three-quarters of mothers knew the purpose of routine immunisation; to prevent childhood illnesses. This is similar to findings in some parts of Ethiopia [14]. More than 70 % of the mothers knew the correct age at which the different vaccines were given with a higher proportion knowing the correct age for the first and last doses. This good knowledge level was consistent with other studies in Nigeria and other African countries [9, 14, 15].

In this study, the target coverage rate of 80 % for all antigens was achieved for antigens given at birth. These vaccines (BCG, OPV0, and HBV1) had higher coverage rates when compared with others given after birth. This could be due to the fact that many of the children were delivered in health facilities and obtained these vaccines before leaving the health facilities. This finding is similar to those in south- western Nigeria and Istanbul [10, 15]. The lowest coverage rates were recorded for measles and yellow fever vaccines, reflecting poor compliance with vaccines administered late in infancy. This finding corroborates with that in Oyo State Nigeria [16]. A plausible reason is the relatively long period between the first dose of vaccines and those of measles and yellow fever which may make mothers forget to return for appointments for these vaccines, moreover when concerns about child care and survival may not be as high as those of the newborn.

Our study revealed that 74.4 % of children were fully immunised by mothers recall alone, while only 57.9 % were fully immunised by vaccination card; this difference could be as a result of inability of mothers to recall actual doses of vaccines and social desirability biases which may lead to overestimation of vaccines received by the children.

In identifying the determinants of complete immunisation, our study relied on card assessment. This was because when we compared the mothers’ assessment of completeness of vaccination with card assessment using Kappa’s statistics, the number of observed agreements was low. This increased the accuracy of our findings as suggested by some authors that relied on mothers’ assessment of vaccination status [9, 17]. About 4 % of children in this district had never been vaccinated based on reports given by their mothers. However, the true proportion in the community was likely to be higher. This finding highlights the importance of tracing and identifying missed children, especially in the final push to eradicate poliomyelitis as clusters of unimmunised children may prevent the interruption of wild polio virus.

Maternal health care utilization indices such as mother’s attendance of antenatal care and maternal tetanus toxoid immunisation status were found to be predictors of completion of immunisation schedule; these factors have been previously demonstrated by several other studies [14, 18–20]. These findings suggest the need to sensitize women to seek health care services during pregnancy and also promote utilisation following childbirth.

In addition, several studies have demonstrated that maternal knowledge on vaccines and routine immunisation improves vaccination uptake [14, 21]. This study also identified this association, as mothers with good knowledge of routine immunisation were found to be twice as likely to fully immunise their children. The finding that having access to information on routine immunisation influences uptake is pertinent as it emphasizes the fact that information reinforces knowledge which eventually leads to utilisation and uptake of health services including immunisation. This finding had also been previously demonstrated in a similar study in Uganda [20].

Geographical accessibility to health facilities offering routine immunisation services is known to be a major determinant of immunisation coverage in many areas of Nigeria and most parts of sub-Saharan Africa, as demonstrated by other studies [18, 20]. However, similar to findings by Jackrati et al. in Mozambique, our study did not also find this association [22]. Many studies have also demonstrated the association between place of delivery of child and immunization uptake [14, 16]. However, this study did not find a significant association between these two factors. It is possible that the health facilities in these areas were not actively promoting vaccination of children after delivery but our study could not verify this assertion because we did not explore health system factors.

One of the strength of this study is that the study was community-based. Data were collected following household visits by trained community health workers ensuring accuracy of information given. Also, the very high vaccination card retention rate of 63.3 % which was higher than reported in the past as national average of 26.1 % [8] gave a fairly accurate assessment on the immunisation coverage in this district.

However the study has some limitations. Firstly, the effect of health system factors as well as immunisation service delivery related factors including vaccine availability, health care personnel and logistics which are known to have an influence on uptake of immunisations were not explored. Secondly, the socioeconomic and paternal factors that may influence completion of immunisation were not assessed. However despite these limitations, the study adds to the body of knowledge on immunisation uptake in rural areas and the results can be generalised to other communities in southwestern Nigeria. Thus it provides useful information to program managers and policy makers to improve immunisation coverage and thereby reduce childhood morbidity and mortality.

## Conclusions

The full immunisation coverage rate in the rural district of Osun State was below the target of at least 80 % of all antigens by 12 months of age. The factors that determined full immunisation status included mothers’ attendance of antenatal care, mothers’ receiving tetanus toxoid immunisation, having access to routine immunisation information as well as having good knowledge on routine immunisation. We recommend that effort should be made to increase maternal health care utilisation such as antenatal care utilisation and uptake of tetanus toxoid immunisation by mothers. Health care providers need to conduct health education activities on the benefits and schedule of immunisation to mothers, to improve their knowledge on routine immunisation.

## Additional file

Additional file 1: Data set on immunisation coverage survey in Atakumosa west LGA, Osun State Nigeria. (XLS 1246 kb)

## Abbreviations

ANC: Antenantal care
BCG: Bacille Calmette Guerin
DEFF: Design effect
DPT: Diptheria-Pertusis-Tetanus
EPI: Expanded program on immunisation
HBV: Hepatitis B vaccine
OPV: Oral polio vaccine
RI: Routine immunisation
TT: Tetanus toxoid
VPD: Vaccine preventable diseases
WHO: World Health Organisation
